# Molecular Diagnostic and Prognostication Assays for the Subtyping of Urinary Bladder Cancer Are on the Way to Illuminating Our Vision

**DOI:** 10.3390/ijms23105620

**Published:** 2022-05-17

**Authors:** Thorsten H. Ecke, Florence Le Calvez-Kelm, Thomas Otto

**Affiliations:** 1 Department of Urology, HELIOS Hospital Bad Saarow, 15526 Bad Saarow, Germany; 2 Department of Urology, Universitätsmedizin Berlin-Charité, 10098 Berlin, Germany; 3 International Agency for Research on Cancer (IARC), 69372 Lyon, France; lecalvezf@iarc.fr; 4 Department of Urology, Rheinland Klinikum Neuss, 41464 Neuss, Germany; thomas.otto@rheinlansklinikum.de; 5 University Hospital Essen, 45147 Essen, Germany

After the successful publication of three Special Issues devoted to highlighting novel scientific research results in the field of bladder cancer and their clinical implications, we are now directing our efforts towards a fourth edition which will aim at compiling innovative research strategies that will ultimately guide and support clinicians in the decision-making process for targeted bladder cancer therapies.

Urothelial carcinoma is a fascinating tumor type characterized by marked tumor heterogeneity. This heterogeneity poses particular challenges for routine clinical practice and research. Research on urothelial carcinoma is currently speeding up—much more than in the years before. Methodological developments achieved in recent years, particularly in the area of high-throughput analyses, have contributed to a better understanding of the biology and heterogeneity of urothelial carcinoma, which has led to the development of new biomarkers and approaches for targeted therapy. Accordingly, this fourth edition includes many new chapters on molecular characterization, urothelial carcinogenesis and potential clinical applications.

Cystoscopy and imaging systems are still considered the gold standard for the detection and monitoring of bladder cancer as they have shown unequal combined overall sensitivity and specificity. However, they still have a limited sensitivity in detecting small lesions of the urinary tract. For this reason, urine cytology is still the most widely used complementary non-invasive test for the detection and surveillance of bladder cancer. Despite its high specificity of around 86%, the limitation of this method lies in its low sensitivity of approximately 50% [1], especially in detecting low-grade tumors [2,3]. Moreover, subjectivity and lack of uniformity in reporting the results of cytology examinations still exist despite the recent effort to better classify urine cytology results as per the Paris System guidelines [4]. Urine biomarkers have been developed and commercialized, but due to performance inconsistences or cost considerations, none of them have been recommended by international guidelines for bladder cancer clinical management [5,6]. Therefore, to date, with the absence of reliable cost-effective urinary biomarkers, the confirmation of suspected carcinomas of the urinary tract and the subsequent life-long surveillance for relapse are still being undertaken by cystoscopic examinations, which represents a significant cost burden on healthcare systems [7].

In contrast to other urine markers, cytology is still recommended in bladder cancer diagnosis and surveillance for recurrence [8]. However, the development of nomograms integrating clinical parameters and potential promising urinary-based tumor markers could, in theory, provide a cost-effective alternative to cystoscopy, therefore improving clinical management during primary detection of bladder cancer and follow-up [9].

Novel markers or combinations of existing tumor markers could significantly contribute to more precise diagnosis and tumor subclassifications as well as facilitating therapeutic decision making. The classification of bladder tumors based on grade and stage alone is suboptimal in predicting the biological behavior and in guiding the choice of treatment, especially in high-risk cases [10,11,12].

The molecular subtyping of bladder cancer based on its transcriptional features has been well characterized after its initial introduction in 2014 [13,14,15]. In particular, muscle-invasive tumors have been categorized into basal and luminal subtypes such as the molecular breast cancer subtypes originally described by Perou et al. [16], which have been subsequently shown to be predictive of clinical outcomes. In this line, basal types of muscle-invasive bladder cancer have been associated with shorter disease-specific and overall survival, presumably because patients with these cancers tended to have more invasive and metastatic disease at presentation [14].

The Lund study included both NMIBC and MIBC and described five expression subclasses with some presenting features that overlapped with luminal and basal subtypes of MIBC [17]. The UROMOL study, which focused on NMIBC only, described three subclasses (some overlapping features of the Lund classes) that showed prognostic significance [18]. There are now three accepted classifications that use different nomenclatures for bladder tumors, and it is critical to evaluate which signatures provide the best clinical utility, especially knowing that they appear to be promising for targeted therapies as some have shown differences in biological behavior and chemotherapy sensitivity [19]. In addition, as transcriptional profile similarities have been observed between bladder cancer and breast cancer subtypes, for which targeted therapies are well established [14,15], these therapeutic approaches may be successfully applied to treat specific bladder cancer patients. For instance, protein expressions of markers for basal (CK5/6, CK14, CD 40) and luminal subtypes (CK20, GATA3, ERß, Uroplakin II, HER2/neu, FGFR3) have been identified.

In addition to transcriptional molecular subtypes, DNA methylation profiles and copy number alterations have defined bladder cancer subtypes that may have some relevant prognostic implications. It is therefore expected that a multi-omics integrated approach could refine the molecular subtyping of bladder cancer, eventually providing the best clinical relevance [20]. Innovative research strategies should also help unveil unclear bladder cancer mechanisms. In a case report of an atypical bladder cancer patient initially presenting with a low-grade tumor that evolved to metastasis and subsequent death, “postmortem” pathological and molecular data were used to better characterize the subtype that could explain the poor prognosis and identify potential targets in critical pathways that may have led to better directed therapies and improved prognosis [21]. Altogether, this shows how research projects integrating molecular characterization of bladder tumors contribute to refining our understanding of bladder carcinogenesis that is essential to develop prognostication assays and target therapies. Early molecular analysis of bladder tumors will enable clinicians to choose the most adapted treatment and timely monitor treatment response and adapt it in case of an absence or incomplete response, for example, via dual blockade of FGFR and ERBB signaling.

In conclusion, there is an urgent and tremendous need for clinical markers that can reliably predict the recurrence and progression of bladder cancer; these markers will likely contribute to establishing better personalized treatments. Molecular staging of urological tumors will allow selecting cases that will require systemic and/or target treatments [22,23].

The editors thank all submitting authors for their efforts and time spent on each manuscript. The lead editor would like to thank all editors for the time spent in reviewing, assigning reviews and commenting on the submitted manuscripts. As the editorial team, we hope that this Special Issue will prove useful in planning future bladder cancer research studies.
